# Identification of a cluster-situated activator of oxytetracycline biosynthesis and manipulation of its expression for improved oxytetracycline production in *Streptomyces rimosus*

**DOI:** 10.1186/s12934-015-0231-7

**Published:** 2015-04-02

**Authors:** Shouliang Yin, Weishan Wang, Xuefeng Wang, Yaxin Zhu, Xiaole Jia, Shanshan Li, Fang Yuan, Yuxiu Zhang, Keqian Yang

**Affiliations:** Department of Environmental and Biological Engineering, School of Chemical and Environmental Engineering, China University of Mining and Technology (Beijing), D11 Xueyuan Road, Haidian District, Beijing, 100083 People’s Republic of China; State Key Laboratory of Microbial Resources, Institute of Microbiology, Chinese Academy of Sciences, 1 Beichen West Road, Chaoyang District, Beijing, 100101 People’s Republic of China; Shengxue Dacheng Pharmaceutical Co., Ltd, 50 Shengxue Road, Shijiazhuang, 051430 Hebei People’s Republic of China

**Keywords:** Oxytetracycline, Activator, SARP, Rational engineering, *Streptomyces rimosus*

## Abstract

**Background:**

Oxytetracycline (OTC) is a broad-spectrum antibiotic commercially produced by *Streptomyces rimosus*. Despite its importance, little is known about the regulation of OTC biosynthesis, which hampered any effort to improve OTC production via engineering regulatory genes.

**Results:**

A gene encoding a *Streptomyces* antibiotic regulatory protein (SARP) was discovered immediately adjacent to the *otrB* gene of *oxy* cluster in *S. rimosus* and designated *otcR*. Deletion and complementation of *otcR* abolished or restored OTC production, respectively, indicating that *otcR* encodes an essential activator of OTC biosynthesis. Then, the predicted consensus SARP-binding sequences were extracted from the promoter regions of *oxy* cluster. Transcriptional analysis in a heterologous GFP reporter system demonstrated that OtcR directly activated the transcription of five *oxy* promoters in *E. coli*, further mutational analysis of a SARP-binding sequence of *oxyI* promoter proved that OtcR directly interacted with the consensus repeats. Therefore, *otcR* was chosen as an engineering target, OTC production was significantly increased by overexpression of *otcR* as tandem copies each under the control of strong SF14 promoter.

**Conclusions:**

A SARP activator, OtcR, was identified in *oxy* cluster of *S. rimosus*; it was shown to directly activate five promoters from *oxy* cluster. Overexpression of *otcR* at an appropriate level dramatically increased OTC production by 6.49 times compared to the parental strain, thus demonstrating the great potential of manipulating OtcR to improve the yield of OTC production.

**Electronic supplementary material:**

The online version of this article (doi:10.1186/s12934-015-0231-7) contains supplementary material, which is available to authorized users.

## Background

Oxytetracycline (5′-hydroxytetracycline; OTC) and related tetracyclines are potent inhibitors of bacterial protein synthesis with broad-spectrum activity against both gram-positive and gram-negative pathogens [1]. Although the clinical use of the tetracyclines has declined in recent years due to their side effects and reduced efficacy, OTC had found wide application in animal feeds and aquaculture, with annual production exceeding 5,000 ton.

OTC is an aromatic polyketide antibiotic commercially produced by *Streptomyces rimosus*. The OTC biosynthetic gene cluster is located in a 34 kb segment of *S. rimosus* genome flanked by two putative resistance genes (*otrA* and *otrB*) [2]. Initially, several genes from the cluster were partially characterized, i.e. genes encoding minimal PKS (*otcY1-1*, *otcY1-2*, and *otcY1-3*) [3], cyclase (*otcD1*) [4], and anhydrotetracycline oxygenase (*otcC*) [5,6]. However, the complete sequence of the OTC biosynthetic gene cluster was not released to the public, so in 2006, Tang’s group systematically re-sequenced the OTC biosynthetic gene cluster and re-named the cluster *oxy* [7]. Afterward, Tang’s group further characterized OTC biosynthetic pathway, by studying several key biosynthetic enzymes, such as the amidotransferase OxyD and the thiolase OxyP for the synthesis of the malonamate starter unit [7,8], the ancillary oxygenase OxyE for more efficient C-4 hydroxylation [9] and two redox enzymes OxyS and OxyR for final transformation of anhydrotetracycline to OTC [10]. However, for the regulation of OTC biosynthesis, there is a paucity of curated information, only one annotated regulatory gene *otrR* was reported which is divergently transcribed from the resistance gene *otrB* [11]. OtrR is a MarR family regulator, but the role of OtrR in OTC production had not been investigated [11]. Based on its location, it could be involved in the regulation of *otrB*, which encodes a MFS family efflux pump probably involved in the export of OTC [11].

Over the years, further clues on the regulation of OTC biosynthesis had emerged. McDowall et al. [12] reported that the sequences in the promoter regions of *otcC*, *otcX*, and *otcY* contain tandem repeats, that are similar to the DNA-binding sites of *Streptomyces* antibiotic regulatory proteins (SARP) transcription activators, with this information they proposed that OTC production could be controlled by an unknown SARP-like activator. Besides, when expressing the OTC cluster in the heterologous host *Streptomyces coelicolor* CH999, Wang et al. [13] found that the SARP regulator, encoded by *ctc*11(*ctcB*) from the chlorotetracycline (CTC) gene cluster (*ctc*) of *Streptomyces aureofaciens* [14], could activate the transcription of *oxy* cluster in heterologous host. Since OTC and CTC are structurally similar antibiotics, they should share similar biosynthetic and regulatory mechanisms. The fact that a SARP regulator (Ctc11) exists in the CTC cluster and Ctc11 could activate *oxy* cluster in the heterologous host strongly implies that a SARP-like regulator may exist in the vicinity of *oxy* cluster. Therefore, in this work, our goal is to identify the SARP-like activator of *oxy* cluster and engineer it to improve OTC production.

Engineering the regulation of antibiotic biosynthesis is an effective strategy to improve antibiotic production [15]. Simple overexpression of pathway-specific activator gene using a constitutive promoter was often used to improve antibiotic production [16]. This simple genetic engineering has been successful in some cases [16,17], but can fall short to optimize the expression of target gene to get the best production [18]. In the era of synthetic biology, fine-tuning gene expression to achieve optimal level of target production has been accepted as key to successful engineering practice [18,19]. Therefore, as a prelude to an engineering attempt, in this work, a series of experiments were performed to determine the optimal expression level of the newly identified activator (OtcR) to give the best yield improvement.

In this work, a cluster-situated SARP-like regulator, OtcR, was discovered immediately adjacent to the resistance gene *otrB* in the upstream of *oxy* cluster. OtcR was confirmed to be a pathway specific activator of OTC biosynthesis, and overexpression of OtcR at an appropriate level significantly increased the production of OTC.

## Results

### Discovery of SARP regulator OtcR

The SARP regulator Ctc11 from the *ctc* gene cluster of *S. aureofaciens* was found to activate the expression of *oxy* cluster in heterologous host *S. coelicolor* CH999 [13], which strongly suggests that the expression of *oxy* cluster in *S. rimosus* is also positively regulated by a native SARP regulator. To find the native SARP regulator of *oxy* cluster in *S. rimosus*, a fosmid library of *S. rimosus* was constructed. First, neighboring genes, up- and down-stream of *oxy* cluster were targeted and sequenced. As predicted, a SARP regulator was identified immediately upstream of *otrB*, and designated *otcR* (Additional file 1: Figure S1A). The complete DNA sequence of *otcR* has been deposited in GenBank under the accession number KP035101. The amino acid sequence of OtcR is closely related to Ctc11 (sharing 46% identities, Additional file 1: Figure S1B). They both have an N terminal DNA-binding domain and a C terminal bacterial transcriptional activation domain (BTAD), but lack the other extra domains usually found in members of the SARP regulators, such as an ATPase domain or tetratricopeptide repeat (TPR) domain [20,21].

### OtcR is essential for OTC production

**Figure 1:**
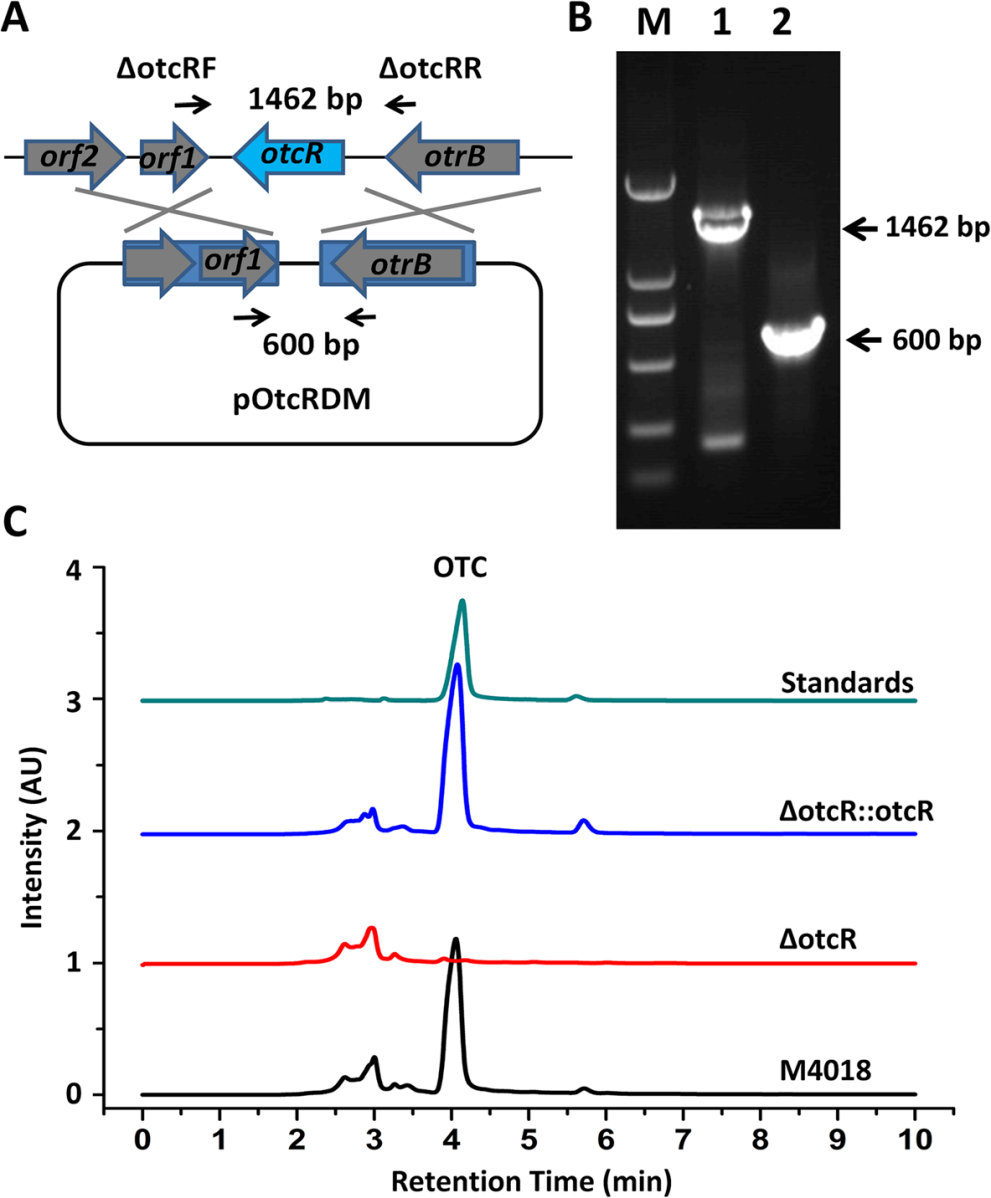
**OtcR is essential for OTC production. A**. Schematic representation of the strategy used for the disruption of *otcR* in *S. rimosus* M4018. The solid arrows indicate the positions of primers used for the confirmation of the disruption mutants (ΔotcR). **B**. PCR verification of the ΔotcR mutant. Line M: marker; line 1: PCR products (1462 bp) using the genome of *S. rimosus* M4018 as template; line 2: PCR products (600 bp) using the genome of ΔotcR as template. **C**. OTC production profiles of *S. rimosus* M4018, ΔotcR and ΔotcR::otcR were analyzed by HPLC (UV 350 nm).

To determine the role of *otcR* in OTC biosynthesis, an *otcR* disruption mutant was constructed via double-crossover recombination (Figure 1A). The correct construction of the mutant (ΔotcR) was confirmed by PCR (Figure 1B). Then ΔotcR was cultured in FM medium to see its effect on OTC production. As expected, in comparison with parental strain *S. rimosus* M4018, OTC production was completely abolished in ΔotcR (Figure 1C). Complementation of ΔotcR with a single copy of *otcR* driven by its own promoter (ΔotcR::otcR) restored the production of OTC to a similar level to the parental strain (Figure 1C). To exclude the possibility of growth effect on OTC production, the biomasses of *S. rimosus* M4018, ΔotcR, and ΔotcR::otcR strains were determined simultaneously, the results showed that the three strains grew similarly (Additional file 2: Figure S2), so the positive effect on OTC production was completely attributable to the function of *otcR*.

### Consensus sequences of the *oxy* promoter regions

**Figure 2:**
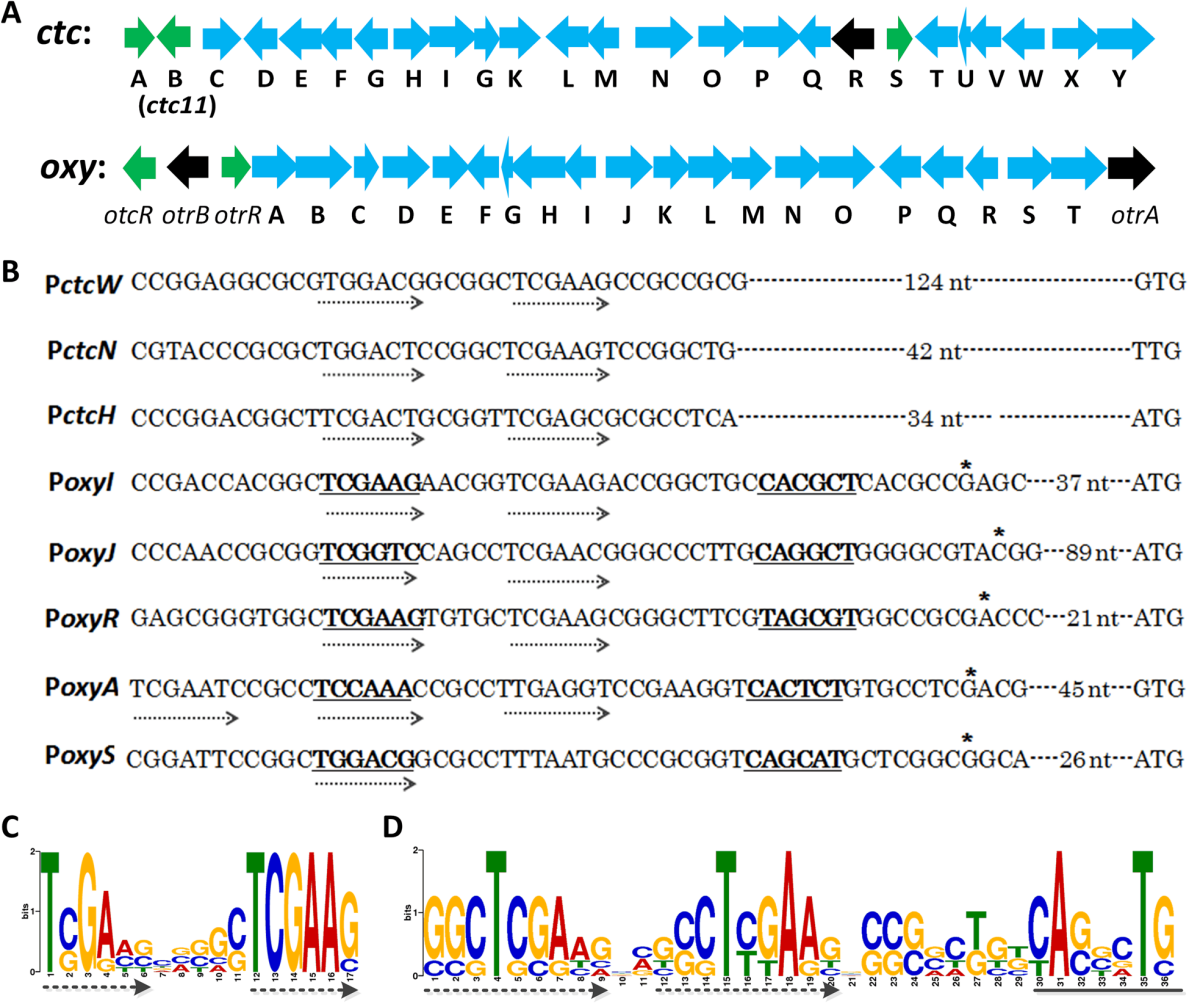
**The predicted SARP**-**binding sites of** ***oxy*** **and** ***ctc*** **clusters. A**. Organization of *ctc* cluster from *S. aureofaciens* and *oxy* cluster from *S. rimosus.* **B**. The predicted SARP-binding consensus sequences of *ctc* and *oxy* clusters. The dotted arrows mark the direct repeat sequences identified in this study. Putative -10 and -35 regions are underlined and indicate with bold letters. **C**. Analysis of the consensus SARP- binding sequences of the promoter regions from *ctc* and *oxy* using MeMe. The dotted arrows mark the direct repeat sequences. **D**. Analysis of the SARP- binding consensus sequences of the promoter regions from *oxy.* The dotted arrows mark the direct repeat sequences identified by MeMe. The conserved consensus sequence of -10 regions was underlined.

Previously, McDowall et al. observed the tandem repeats recognized by SARP-like regulator in the *otcC*, *otcX*, and *otcY* promoter regions [12]. To examine the presence of SARP-binding tandem repeats in *oxy* promoters, the potential consensus sequence motif of *oxy* promoter regions was extracted; the promoter regions of *ctc* were simultaneously analyzed because the SARP regulator Ctc11 could activate the expression of *oxy* cluster. The sequences of *ctc* and *oxy* were obtained from GenBank (accession numbers HM627755 [22] and DQ143963 [7], respectively, Figure 2A). As the divergent intergenic regions in both clusters should contain bidirectional promoters, the sequences of these regions were aligned. As shown in Figure 2B, two or three direct hexameric repeats were identified in the promoter regions of *ctcW*, *ctcN*, *ctcH*, *oxyI*, *oxyJ*, *oxyR*, *oxyS* and *oxyA* (Figure 2B). Then the consensus sequence of these repeats was calculated by MeMe [23], which displayed the same two hexameric repeats as the results of alignment (Figure 2C). It is important to note that the identified repeats are separated by 11 bp, corresponding to one complete turn of DNA helix. It is a typical feature of the binding sites of SARP regulators, as SARPs normally form homodimers *in vivo* and are expected to bind two direct repeats at the same side of DNA [21].

To further define the consensus SARP-binding motif of the *oxy* promoters, the promoter regions of *oxy* (*oxyI*, *oxyJ*, *oxyR*, *oxyS* and *oxyA*) were analyzed by MeMe separately. To our surprise, the consensus motif of *oxy* showed a little difference from that of *ctc* cluster, which included two 9 nt direct repeats (Figure 2D). The forward 9 nt repeat is also separated from the adjacent one by 11 bp. It should be noted that the 9 nt direct repeats specific for *oxy* promoters encompass the 6 nt repeats identified from both *oxy* and *ctc* cluster.

### OtcR directly activates *oxy* promoters that contain the consensus repeat sequences

**Figure 3:**
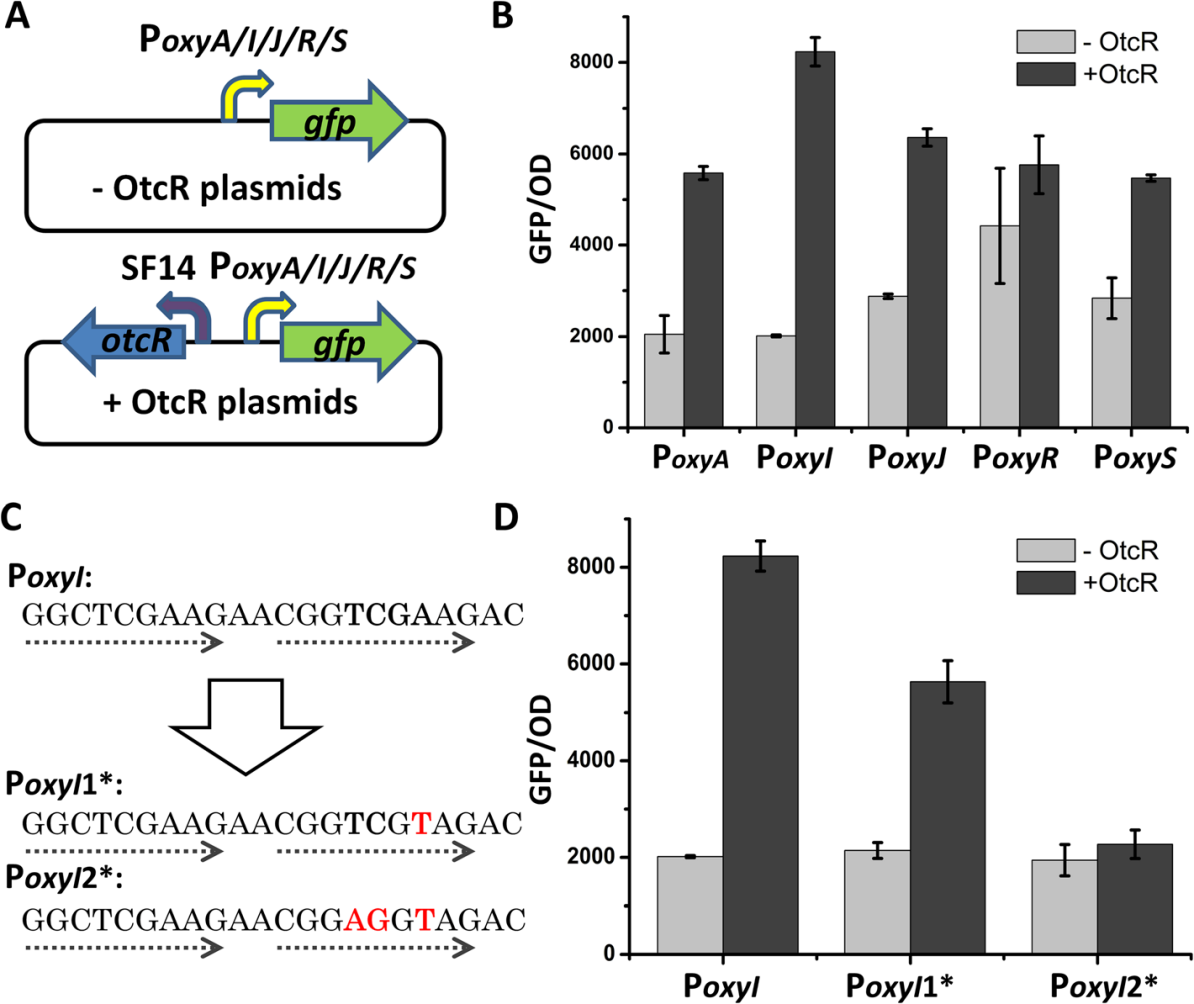
**OtcR directly activates the transcription from** ***oxy*** **promoters** ***in vivo*** **. A**. An illustration of the reporter plasmids. **B**. Detection of the regulatory relationship of OtcR and *oxy* promoters using *gfp* reporter plasmids in *E.coli*. **C**. Sequences of the direct repeats of native *oxyI* promoter (P*oxyI*) and two mutants (P*oxyI*1* and P*oxyI*2*). **D**. Detection of the interaction of OtcR with the direct repeat sequences of P*oxyI*, P*oxyI*1* and P*oxyI*2* using *gfp* reporters. The values are means ± SD from three independent experiments.

To determine whether OtcR directly interacts with the promoter regions of *oxyA*, *oxyI*, *oxyJ*, *oxyR* and *oxyS*, a straight forward experiment to perform would be electrophoretic mobility shift assays (EMSA) of OtcR with these regions *in vitro*. However, soluble expression of recombinant OtcR in *E. coli* failed despite considerable effort. Therefore, we designed a reporter system in *E. coli* to demonstrate the regulatory relationship. As shown in Figure 3A, plasmids, in which *gfp* gene was directly controlled by promoters of *oxyA*, *oxyI*, *oxyJ*, *oxyR* and *oxyS* respectively, were transformed into *E. coli*. The resulting strains were endowed with the abilities to emit green fluorescence (Figure 3B).When *otcR* driven by SF14 promoter was inserted into the above plasmids (Figure 3A) and transformed into *E. coli*, fluorescences were enhanced ranging from 1.3 to 4 folds compared to that without *otcR* (Figure 3B). These results indicated that OtcR indeed activates the expression of *oxy* promoters in a heterologous *E. coli* host.

To further verify whether the regulatory relationship is due to the interaction of OtcR with the 9 nt direct repeats, we mutated the second 9 nt direct repeats (CGGTCGAAG) of *oxyI* promoter (P*oxyI*) to CGGTCGTAG (P*oxyI*1*) and CGGAGGTAG (P*oxyI*2*), respectively (Figure 3C), and tested the effects of OtcR on the two mutant promoters using above reporter system. When OtcR was not expressed, the two mutant promoters showed similar activities as P*oxyI* (Figure 3D). However, when *otcR* was inserted into the reporter plasmids and OtcR was expressed, P*oxyI*1* showed less enhancement of fluorescence than that of P*oxyI*. Remarkably, the activity of P*oxyI*2* was no longer affected by OtcR (Figure 3D).These results indicated that OtcR lost the ability to interact with the mutant promoter P*oxyI*2*, thus had no effect on the transcription from P*oxyI*2*. Based on these results, we conclude that OtcR exerts its activator effect by binding to the 9 nt direct repeats in P*oxyI*. This conclusion should also apply to the other promoters of *oxy* cluster, as the consensus repeat sequences of these promoters share high degree of similarity.

### Overexpression of *otcR*

**Figure 4:**
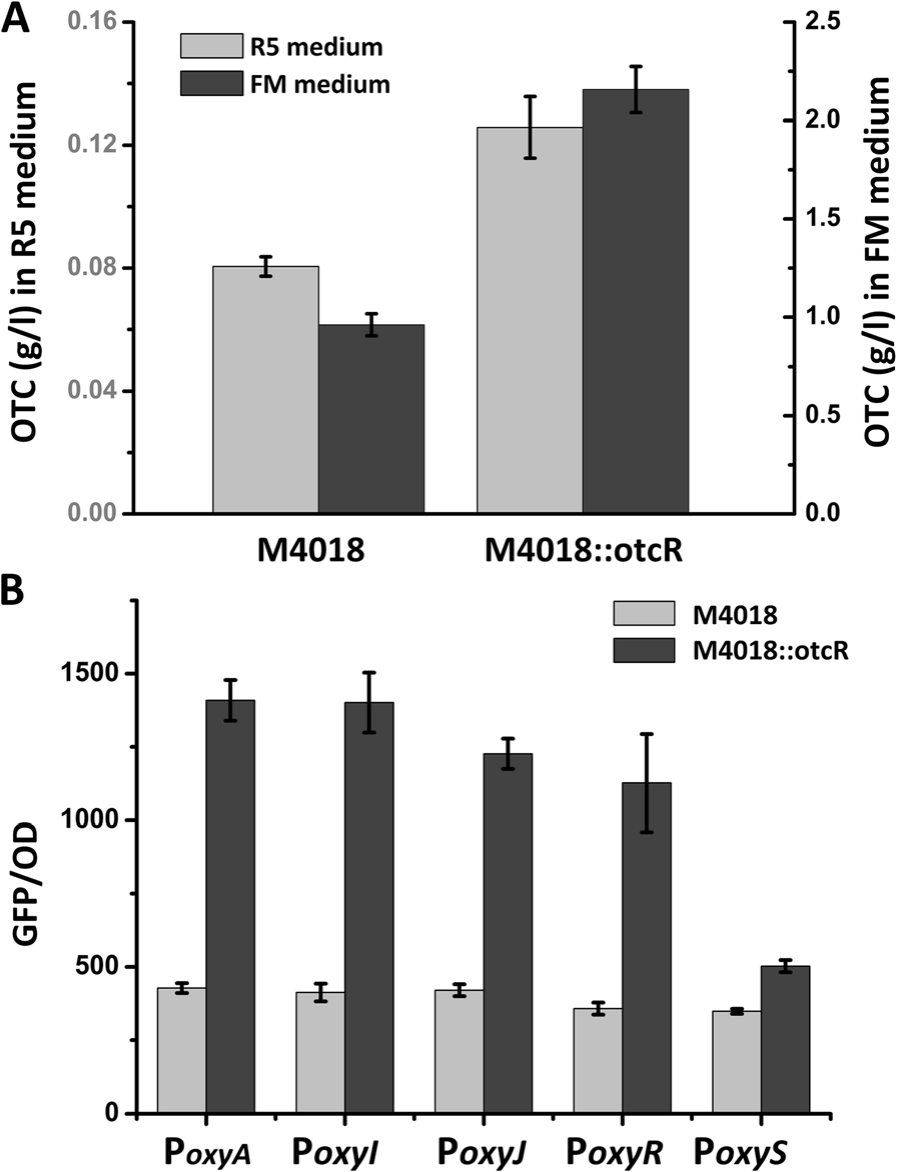
**Overexpression of** ***otcR*** **improves OTC production. A**. OTC production of *S. rimosus* M4018 and M4018::otcR in R5 and FM media. The values are means ± SD from three independent experiments. **B**. Activities of *oxy* promoters in *S. rimosus* M4018 and M4018::otcR reported by GFP. Green fluorescence of different strains was measured after incubation in R5 medium at stationary phase (10 d). The values are means ± SD from three independent experiments.

Since *otcR* encodes a SARP cluster-situated regulator (CSR), positively regulating the expression of OTC biosynthesis, overexpression of *otcR* was adopted as a rational strategy to improve OTC production in *S. rimosus* M4018. An additional copy of *otcR* with its own promoter was cloned in plasmid pOtcR, the plasmid was transformed into M4018 to create M4018::otcR. OTC production of M4018::otcR increased significantly compared to that of M4018 (Figure 4A), both in R5 and FM media. To verify whether the improvement of OTC production was due to enhanced expression of *oxy* cluster, the activities of *oxy* promoters were examined using GFP reporter in R5 medium. As shown in Figure 4B, green fluorescences conferred by the promoters of *oxy* were much higher in M4018::otcR than those in M4018, indicating that the overexpression of *otcR* indeed improved the transcription level of *oxy* genes. Thus, the overexpression of *otcR* is a viable strategy to improve OTC production in 4018.

### Improvement of OTC production by manipulating the expression levels of *otcR*

**Figure 5:**
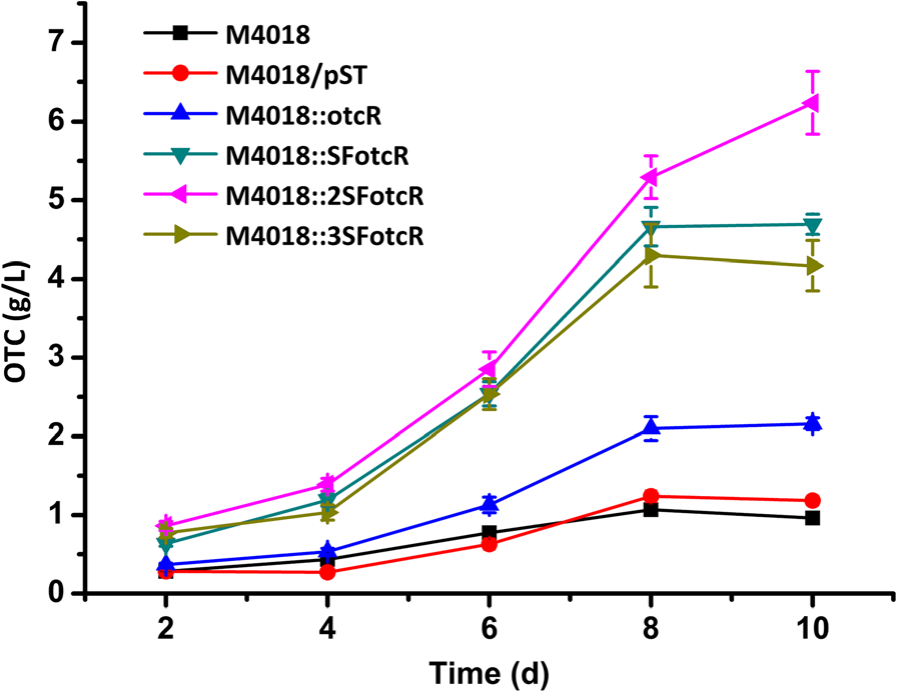
**OTC production profiles of the engineered** ***otcR*** **overexpression strains** ***.*** M4018/pST contains the control plasmid integrated in the genome of M4018; M4018::otcR contains an integrated copy of *otcR* driven by its own promoter; M4018::SFotcR, M4018::2SFotcR and M4018::3SFotcR contain one, two or three integrated copies of *otcR* driven by SF14 promoters. The values are means ± SD from three independent experiments.

As shown above, overexpression of *otcR* is an effective strategy to increase OTC production. To detect the optimum expression level of *otcR*, we designed new expression plasmids to manipulate the expression levels of *otcR.* In a first attempt, *otcR* was cloned in an integrative vector under the control of a strong constitutive promoter SF14 [24], and the plasmid was transformed and integrated into the chromosome of *S. rimosus* M4018 to create M4018::SFotcR. The OTC production of M4018::SFotcR was further increased in comparison with that of M4018::otcR, which contains one integrated copy of *otcR* under the control of its own promoter (Figure 5). Again, the activities of SF14 and *otcR* promoter were quantified by using GFP reporter, indicating that SF14 promoter had stronger transcriptional activity than *otcR* promoter at stationary phase (Additional file 3: Figure S3). These results demonstrated that the expression level of *otcR* is positively correlated with OTC production. To further improve OTC production, plasmids containing two or three copies of *otcR* driven by SF14 promoter were constructed and transformed into 4018 thus creating strains M4018::2SFotcR and M4018::3SFotcR, respectively. As shown in Figure 5, M4018::2SFotcR gave the highest OTC production (6.24 g/l), which is 6.49 times as much as the production of M4018. While the OTC production of M4018::3SFotcR was less than in M4018::2SFotcR and M4018::SFotcR, indicating that the excessive expression of *oxy* genes does not coordinate the physiological condition of the producer.

## Discussion

Productivity improvement of industrially used microorganisms is important to maintain the commercial viability of the corresponding industry. Previously, random mutagenesis in combination with optimization of large-scale industrial fermentations [25,26] and rational metabolic engineering [27-29] have been carried out to improve OTC production in *S. rimosus*. However, due to lack of knowledge about the regulation of OTC biosynthesis, manipulations of regulatory genes to improve OTC production were not reported. Lesnik et al. [30] have identified a LAL (LuxR) family of transcriptional regulator OtcG, which located downstream of the *otrA* gene. OtcG plays a ‘conditionally positive’ role in OTC biosynthesis: inactivation of the *otcG* gene reduced the production of OTC by more than 40 %, but overexpression of *otcG* by introducing a second copy under the constitutive promoter *ermE* did not yield any statistically significant change in OTC production [30]. In this work, a SARP activator, OtcR, was identified in the *oxy* cluster. When we searched for *otcR* in the released genome of *S. rimosus* ATCC 10970 (accession no. ANSJ00000000), a single-base pair insertion mutation was found in the coding sequence of *otcR*. The incomplete sequence (ELQ83266.1) was annotated as SARP family transcriptional regulator as well, which may be the main obstacle of dissecting the regulatory role of OtcR.

SARP regulators are characterized by their DNA-binding domains that resemble that of OmpR, and by an accompanying BTAD [31]. The sequence alignment of promoters regulated by SARPs revealed that the binding sites of these proteins contain common features [21], e.g. two direct repeats, the 3′ repeat locates 8 bp upstream of the -10 element (see Figure 2B), and the 5′ repeat overlaps with the -35 region of target promoter; the two repeats are separated from each other by 11 bp, corresponding to one complete turn of the DNA helix. Consequently, the two repeats of SARP-binding sites appear on the same face of the DNA helix, opposite to the face bound by RNA polymerase [21]. This topology would allow SARPs and RNA polymerase to have simultaneous access to the promoter region [21]. Therefore, to identify the existence of SARP-binding sites, such criteria were applied on the predicted promoter regions of *oxy* and *ctc* clusters. All the promoter regions of *oxy* showed the typical features of SARP-binding site.

SARP regulators, as activators of antibiotic biosynthesis in *Streptomyces*, are often found in the corresponding gene clusters, for example, ActII-ORF4 [32] and RedD [33] were identified in the actinorhodin and undecylprodigiosin gene clusters in *S. coelicolor* A3(2), respectively. Other examples include DnrI from the daunorubicin biosynthetic cluster in *Streptomyces peucetius* [34]; MtmR from the mithramycin biosynthetic cluster in *Streptomyces argillaceus* [35] and CcaR from the cephamycin and clavulanic acid super cluster in *Streptomyces clavuligerus* [36]. Therefore, to search for a SARP-like activator for OTC biosynthesis, the vicinity regions of *oxy* cluster were firstly investigated.

SARP regulators show great diversity in domain organization. The diversity is exemplified by their lengths, which vary from about 300 residues (e.g. ActII-ORF4 and DnrI [32,34]) to 1000 residues (e.g. AfsR, PimR and SanG [21,37,38]). The small SARP proteins usually contain two domains: an N-terminal DNA-binding domain and a C-terminal BTAD. In the cases of large SARPs, an extra ATPase domain and a TPR domain are extended downstream of the BTAD domain, which are involved in sensing endogenous signals (such as ADP/ATP pool) and regulating the activity of SARPs [20,21]. Compared to known SARPs, the length of OtcR is relatively short (251 amino acids), containing only the DNA binding and BTAD domains. Thus we deduce that OtcR is a pathway specific activator of OTC cluster not modulated by other signals. In addition, we were able to demonstrate that OtcR directly activates the expression of five *oxy* promoters *in vivo* and showed that the regulation is achieved by direct interaction of OtcR with the repeat sequence of *oxy* promoters to enhance their expression (Figure 3). These findings gave us confidence to rationally engineer *otcR* expression to improve OTC production in *S. rimosus*.

It is important to mention that the 9 nt direct repeats of *oxy* promoters encompass the 6 nt repeats identified from both *oxy* and *ctc* cluster, which could explain the finding that Ctc11 could activate the transcription of *oxy* in a heterologous host [13]. Based on sequence analyses, Ctc11 (CtcB) and OtcR share 46% identities, in addition, the predicted SARP-binding sites were also identified in *ctc* cluster. Hence, Ctc11 is most likely the activator of *ctc* cluster and an ideal target for the engineering of CTC production.

SARP regulators as activators of antibiotic production have been targeted for engineering to improve the production of corresponding antibiotics in *Streptomyces* [16]. However, simple overexpression of activator genes may not give the best performance, fine-tuning of the expression levels of SARP activator should be tried to coordinate the expression of biosynthetic pathway with the physiology of producers. Recently, Sohoni et al. [18] used a synthetic promoter library to optimize the expression of ActII-ORF4; they discovered that a promoter giving the highest actinorhodin production level has a unique expression profile in term of strength and timing. In this work, *otcR* was overexpressed by the native and SF14 promoters, respectively. Our results showed that SF14 is stronger than the native *otcR* promoter at stationary phase (Additional file 3: Figure S3). As a consequence, the OTC production of M4018::SFotcR was significantly higher than that of M4018::otcR (Figure 5), suggesting higher expression levels of *otcR* are needed to further improve OTC production in *S. rimosus* M4018. So overexpression of *otcR* as tandem copies using SF14 promoter was tested, and further enhancement of OTC production level was observed. However, integrating only two copies of overexpressed *otcR* (M4018::2SFotcR) was found to confer the highest OTC production to M4018, three copies of *otcR* may lead to excessive burden on the cell, thus reducing OTC production. These results further emphasize the need to stepwise calibrate the expression level to determine the optimum expression level of activator genes.

## Conclusions

In this work, OtcR was identified as a cluster-situated pathway specific activator of OTC biosynthesis in *S. rimosus* M4018. Moreover, manipulation of the expression levels of *otcR* could increase OTC production to more than six times in *S. rimosus* M4018. Therefore, engineering the expression of *otcR* is a promising alternative strategy for the engineering of OTC production strains.

## Methods

### Strains, plasmids and culture conditions

Strains and plasmids used in this study are listed in Table 1. *E. coli* TOP10 and DH5α were used for cloning and GFP assay, respectively; *E. coli* ET12567/pUZ8002 was used for conjugation between *E. coli* and *S. rimosus* M4018. *Streptomyces* strains were grown on TSB medium (Oxoid) for genomic DNA isolation. Spores of *S. rimosus* were obtained on MS medium [39] after incubation at 30°C for 5 days. The seed cultures of *S. rimosus* strains for fermentation were obtained after growing in seed medium (3% corn starch, 0.3% soya bean, 0.5% calcium carbonate, 0.4% (NH_4_)_2_SO_4_, 0.5% NaCl, 0.015% KH_2_PO_4_ and 0.4% corn steep liquor) at 30°C for 1 d. For GFP assay, *S. rimosus* strains were grown on R5 medium [39]. For measurement of OTC production, both R5 medium and optimum FM medium (5% corn starch, 2% soya bean, 1.4% calcium carbonate, 1.4% (NH_4_)_2_SO_4_, 0.4% NaCl, 0.01% KH_2_PO_4_, 0.4% corn steep liquor, 0.001% CoCl_2_ and 0.1-0.2% amylase) were used, the fermentation cultures were grown at 30°C for 10 d. *E. coli* strains were grown in Luria-Bertani (LB) containing apramycin (50 μg/ml), kanamycin (25 μg/ml) or chloramphenicol (25 μg/ml) when necessary. *S. rimosus* exconjugants were selected on MS medium plates containing 50 μg/ml nalidixic acid and 500 μg/ml apramycin. High-fidelity PCR was performed using Q5 DNA polymerase (NEB) to obtain DNA fragments used for plasmid construction.

**Table 1:** **Strains and plasmids used in this study**

**Name**	**Description**	**Sources**
***S. rimosus***		
M4018	a strain used for commercial production of OTC	[27]
ΔotcR	*otcR* gene disruption mutant, derived from M4018	This study
ΔotcR::otcR	*otcR* complemented strain, derived from ΔotcR	This study
M4018::otcR	*otcR* overexpressed strain, derived from M4018	This study
M4018/pST	Integrated a control plasmid pST in the genome of M4018	This study
M4018::SFotcR	Integrated a copy of SF14-driven *otcR* in the genome of M4018	This study
M4018::2SFotcR	Integrated two copies of SF14-driven *otcR* in the genome of M4018	This study
M4018::3SFotcR	Integrated three copies of SF14-driven *otcR* in the genome of M4018	This study
***E.coli***		
TOP10	General cloning host for plasmid manipulation	Novagen
ET12567 (pUZ8002)	Donor strain for conjugation between *E. coli* and *Streptomycetes*	[38]
DH5α	Host for reporter system	Novagen
**Plasmids**		
p4-16 h-7 s	Fosmid harbouring entire *oxy* gene cluster	This study
pOtcRDM	For deletion *otcR* gene, containing left and right arms of *otcR*	This study
pGusT-SF14	Derived from pSET152, containing SF14-driven *gusA*	[39]
pST	Derived from pGusT-SF14, deleting the SF14-driven *gusA* in pGusT-SF14	This study
pSF14-otcR	Derived from pGusT-SF14, SF14-driven *otcR*	This study
pSF142-otcR	Derived from pSF14-otcR, containing two copies of SF14-driven *otcR*	This study
pSF143-otcR	Derived from pSF142-otcR, containing three copies of SF14-driven *otcR*	This study
pOtcR	Derived from pGusT-SF14, containing a copy of *otcR* driven by its own promoter	This study
pTAC	Template plasmid containing *gfp*	[40]
pSF14-GFP	Derived from pGusT-SF14, containing SF14-driven *gfp*	This study
pAGFP	Derived from pSF14-GFP, inserting *oxyA* promoter into upstream of *gfp*	This study
pIGFP	Derived from pSF14-GFP, inserting *oxyI* promoter into upstream of *gfp*	This study
pI1*GFP	Derived from pIGFP, inserting *oxyI*1* promoter into upstream of *gfp*	This study
pI2*GFP	Derived from pIGFP, inserting *oxyI*2* promoter into upstream of *gfp*	This study
pJGFP	Derived from pSF14-GFP, inserting *oxyJ* promoter into upstream of *gfp*	This study
pRGFP	Derived from pSF14-GFP, inserting *oxyR* promoter into upstream of *gfp*	This study
pSGFP	Derived from pSF14-GFP, inserting *oxyS* promoter into upstream of *gfp*	This study
pRAGFP	Derived from pAGFP, inserting *otcR* driven by SF14	This study
pRIGFP	Derived from pIGFP, inserting *otcR* driven by SF14	This study
pRI1*GFP	Derived from pI1*GFP, inserting *otcR* driven by SF14	This study
pRI2*GFP	Derived from pI2*GFP, inserting *otcR* driven by SF14	This study
pRJGFP	Derived from pJGFP, inserting *otcR* driven by SF14	This study
pRRGFP	Derived from pRGFP, inserting *otcR* driven by SF14	This study
pRSGFP	Derived from pSGFP, inserting *otcR* driven by SF14	This study
pOtcR-AGFP	Derived from pAGFP, inserting *otcR* driven by its own promoter	This study
pOtcR-IGFP	Derived from pIGFP, inserting *otcR* driven by its own promoter	This study
pOtcR-JGFP	Derived from pJGFP, inserting *otcR* driven by its own promoter	This study
pOtcR-RGFP	Derived from pRGFP, inserting *otcR* driven by its own promoter	This study
pOtcR-SGFP	Derived from pSGFP, inserting *otcR* driven by its own promoter	This study

*The asterisk is used to distinguish the engineered promoter from the native promoter (P*oxyI*).

### Identification of *otcR* from a fosmid library of *S. rimosus*

The genomic DNA library of *S. rimosus* M4018 was constructed using the EpiCentre CopyControl Fosmid Library Production kit in the pCC1FOS vector, according to the manufacturer’s instructions. Briefly, genomic DNA of *S. rimosus* M4018 was size-fractionated in a 0.5% low-melting point agarose gel, and DNA fragments in the 33-48 kb range were collected for library construction. The purified genomic DNA fragments were ligated into a linearized pCC1FOS vector. The ligation mixture was then packaged into lambda phages using packaging extracts, and subsequently introduced into *E. coli* EPI-300. These cells were then plated onto LB agar plates containing 12.5 μg/ml chloramphenicol, and grown overnight aerobically at 37°C, then the *E. coli* colonies were picked and stocked in 384-well-plates. PCR was carried out to screen for clones harboring the *oxy* gene cluster using the primers otc-up-F/otc-up-R and otc-down-F/otc-down-R. The regions upstream and downstream of *oxy* cluster were sequenced by fosmid walking.

The sequence of a fosmid clone (p4-16 h-7 s, Table 1) containing the *oxy* gene cluster were analyzed using Frameplot 4.0 beta (http://nocardia.nih.go.jp/fp4/) and the functions of coding regions were predicted by BLASTp (http://blast.ncbi.nlm.nih.gov/Blast.cgi). A gene encoding a SARP-type activator was found in a region upstream of *oxy* cluster and designated *otcR*. The nucleotide sequence of *otcR* has been submitted to the GenBank (Accession number: KP035101).

### Construction of *otcR* mutant (∆otcR) and complementation mutant (∆otcR::otcR)

To construct *otcR* disruption plasmid pOtcRDM, a 3146 bp fragment upstream of *otcR* was amplified with primers OtcR-up-F2/OtcR-up-R2 (Additional file 4: Table S1); a 3130 bp fragment downstream of *otcR* was amplified with primers OtcR-down-F2/OtcR-down-R2 (Additional file 4: Table S1) from genomic DNA of *S. rimosus* M4018. The two fragments were trimmed by appropriate enzymes and inserted into *Hind*III and *Eco*RI digested pKC1132 [39] to obtain pOtcRDM. The recombinant plasmid was introduced into *E. coli* ET12567 (pUZ8002) and conjugated into *S. rimosus* M4018. The transformants were selected for lower apramycin resistance to get double-crossover mutant (∆otcR) following standard procedures [39]. The correct isolation of ∆otcR was confirmed by PCR amplification using primers ∆OtcR-Test2-F/∆OtcR-Test2-R (Additional file 4: Table S1), in which M4018 gave 1462 bp band, whereas ∆otcR gave a 600 bp band. To construct the complementation plasmid pOtcR, a 1029 bp fragment containing both promoter and *otcR* was amplified from the genomic DNA of M4018 with primers PotcR-F/PotcR-R (Additional file 4: Table S1). The PCR products were double digested with *Bgl*II/*Xba*I and ligated into the corresponding sites of pGusT-SF14 [40] to generate plasmid pOtcR. The plasmid was transformed into ∆otcR by conjugation. The resulting complementation strain (∆otcR::otcR) containing an integrated copy of *otcR* was confirmed by PCR with primers TestF2/TestR2 (Additional file 4: Table S1).

### Demonstration of the influence of OtcR on *oxy* promoters using GFP reporter in *E.coli*

To assemble reporter plasmids for *oxy* promoters, pGusT-SF14 [40] was digested with *Bam*HI/*Xba*I, and the green fluorescence gene (*gfp*) was amplified from pTAC [41] with primers Gfp-F/Gfp-R. The two fragments were joined into a covalently sealed molecule using the recombination method described by Gibson et al. [42] to generate pSF14-GFP. The promoters of *oxyA*, *oxyI*, *oxyJ*, *oxyR* and *oxyS* were amplified with primers PoxyA-gfp-F/PoxyA-gfp-R, PoxyI-gfp-F/PoxyI-gfp- R, PoxyJ-gfp-F/PoxyJ-gfp-R, PoxyR-gfp-F/PoxyR-gfp-R and PoxyS-gfp-F/PoxyS-gfp-R (Additional file 4: Table S1), respectively. The promoterless pSF14-GFP fragments were obtained by *Bgl*II/*Bam*HI double digestion. Using the same assembly method, the promoters of *oxyA*, *oxyI*, *oxyJ*, *oxyR* and *oxyS* were jointed with the promoterless pSF14-GFP fragments to create the reporter plasmids pAGFP, pIGFP, pJGFP, pRGFP and pSGFP, respectively. To evaluate the regulatory effect of *otcR* on *oxy* promoters, the SF14 promoter and *otcR* fragments were amplified from pGusT-SF14 and pOtcR with primers SF14-F/SF14-R and Gib-otcR-F1/Gib-otcR-R1 (Additional file 4: Table S1), respectively. The plasmids pAGFP, pIGFP, pJGFP, pRGFP and pSGFP were digested by *Nhe*I and assembled with the above two fragments to obtain the corresponding reporter plasmids pRAGFP, pRIJFP, pRJGFP, pRRGFP and pRSGFP, respectively, which contain both an *otcR* under the control of SF14 promoter and a *gfp* controlled by *oxy* promoters, the two genes were placed in the opposite orientations (Figure 3A). These plasmids were transformed into DH5α to detect green fluorescence (excitation at 485 nm; emission at 510 nm, Synergy H4 Multi-Mode Reader). All fluorescence values were normalized to growth rates (OD_600_). Each value and error bar represents the average and standard deviation of three experimental replicates, respectively.

### Verification of the interaction between OtcR and the direct repeat of *oxyI* promoter

The promoter of *oxyI* (P*oxyI*) was chosen as a representative to investigate the interaction between OtcR and the predicted SARPs-binding direct repeats. To replace P*oxyI* by P*oxyI*1* in pIGFP and pRIGFP, primers PoxyI1*F/PoxyI1*R were used to amplify the linear fragments using pIGFP and pRIGFP as a template, respectively. Then these two PCR products were self-ligated to create pI1*GFP and pRI1*GFP, respectively. Using the same strategy, primers PoxyI2*F/PoxyI2*R were applied to generate pI2*GFP and pRI2*GFP linear fragments and the fragments were self-ligated to obtain pI2*GFP and pRI2*GFP plasmids. These plasmids were transformed into DH5α and green fluorescence was detected as above.

### Construction of *otcR* overexpression strain M4018::otcR

The complementation plasmid pOtcR was transformed into M4018 by conjugation to generate M4018::otcR, in which pOtcR was integrated in the chromosome of M4018. The control plasmid pST was obtained by self-ligation of a 5632 bp fragment of pGusT-SF14 after digestion by *BamH*I and *Xba*I; pST was transformed into M4018 by conjugation, and integrated into the chromosome of M4018 to generate M4018/pST as control.

To test the activities of *oxy* promoters in M4018::otcR, a fragment containing both *otcR* and its native promoter was amplified with primers Gib-PotcR-F/Gib-PotcR-R (Additional file 4: Table S1) using pOtcR as template. The PCR product was inserted into the *Nhe*I site of pAGFP, pIJFP, pJGFP, pRGFP and pSGFP to generate plasmid pOtcR-AGFP, pOtcR-IGFP, pOtcR-JGFP, pOtcR-RGFP and pOtcR-SGFP, respectively. These plasmids were transformed into M4018 to obtain the corresponding strains in which the plasmids were integrated into the chromosome of M4018, these strains could report the activities of *oxy* promoters in M4018::otcR (containing 2 copies of *otcR*). As controls, pAGFP, pIJFP, pJGFP, pRGFP and pSGFP were also transformed into M4018, the integration of these plasmids into the chromosome of M4018 generated strains that could report the activities of *oxy* promoters in M4018 from its single native copy of *otcR*. For green fluorescence detection, 200 μl stationary phase (10 d) fermentation cultures were assayed as above. All fluorescence values were normalized to the biomass determined by diphenylamine colorimetric method [43]. Each value and error bar represents the average and standard deviation of three experimental replicates, respectively.

### Fermentation and OTC detection

A spore suspension (1.0 × 10^8^) was inoculated into 40 ml of seed medium and grown for 24 h. Then, 4 ml of the first seed culture was inoculated into 40 ml of optimum FM medium or R5 medium. All fermentation cultures were grown at 250 rpm, 30°C for 10 days.

To quantify the production of OTC, the fermentation culture samples were adjusted to pH1.5-2.0 with 9 M HCl, and 1 ml cultures were centrifuged at 12,000 rpm for 10 min. Then, the samples were subjected to HPLC analysis on a Shimadzu Prominence HPLC system with dual λUV detector and YMC polymer C18 column (4.6 × 250 mm). Separation was performed at the following conditions: 60% H_2_O, 10% methanol, 20% acetonitrile and 10% phosphoric acid (2 mM) with a constant flow rate of 1 ml/min. The corresponding peak areas detected at 350 nm were used to calculate the concentration of OTC.

### Overexpression of *otcR* at different levels in *S. rimosus* M4018

Plasmids that will allow *otcR* to integrate into the chromosome of M4018 one to three copies under the control of constitutive promoter SF14 were constructed. To generate pSF14-otcR, promoterless *otcR* fragment was amplified with primers OtcR-F/OtcR-R (Additional file 4: Table S1) and inserted into *Bam*HI/*Xba*I digested pGusT-SF14. To generate pSF142-otcR, pSF14-otcR was digested by *Xba*I and ligated with the fragment amplified using primers Gib-OtcR-F2/Gib-OtcR-R2 (Additional file 4: Table S1) from pSF14-otcR. pSF143-otcR was constructed similarly by inserting Gib-OtcR-F2/Gib-OtcR-R2 (Additional file 4: Table S1) amplified fragment into pSF142-otcR. All plasmids were transformed into M4018 and the plasmids were integrated into the chromosome of *S. rimosus* M4018.

## Additional files

Additional file 1: Figure S1. Organization of *oxy* cluster in *S. rimosus* (A) and alignment of amino acid sequences between Ctc11 and OtcR (B). Additional file 2: Figure S2. Growth of *S. rimosus* M4018, ΔotcR, ΔotcR::otcR and M4018::otcR in R5 medium. The values are means ± SD from three independent experiments. Additional file 3: Figure S3. Comparison of the activities between the *otcR* (P*otcR*) and SF14 promoter using GFP reporters. The values are means ± SD from three independent experiments. Additional file 4: Table S1. Primers used in this study.
