# Supplementary figures and images for: Identification of a cluster-situated activator of oxytetracycline biosynthesis and manipulation of its expression for improved oxytetracycline production in Streptomyces rimosus
Source: Microb Cell Fact. 2015 Apr 2;14:46. doi: 10.1186/s12934-015-0231-7 (PMC4393881; doi:10.1186/s12934-015-0231-7)

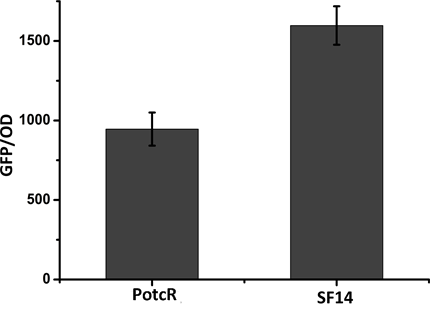

Supplement: Additional file 3: Figure S3. — Comparison of the activities between the otcR (PotcR) and SF14 promoter using GFP reporters. The values are means ± SD from three independent experiments. [file 12934_2015_231_MOESM3_ESM.tif]
