# Supplementary material for: Identification of a cluster-situated activator of oxytetracycline biosynthesis and manipulation of its expression for improved oxytetracycline production in Streptomyces rimosus
Source: Microb Cell Fact. 2015 Apr 2;14:46. doi: 10.1186/s12934-015-0231-7 (PMC4393881; doi:10.1186/s12934-015-0231-7)
Supplement: Additional file 4: Table S1. — Primers used in this study. [file 12934_2015_231_MOESM4_ESM.docx]

**Table S1** - **Primers used in this study.**

| Name | Sequence (5` to 3`) | Use |
| --- | --- | --- |
| otc-up-F | CGAGGACATTCCGGTCGAGG | Clones screening for harboring the whole *oxy* gene cluster |
| otc-up-R | CTGTGATTACGACGCGTCGC |  |
| otc-down-F | CGGTGACTGGCTGATAGTCG |  |
| otc-down-R | CTGGTCTTGCCGGCGTCAAC |  |
| OtcR-up-F2 | CCC*AAGCTT*GTGGCCATTGCGGACTTCTACC (*Hind*III) | Construction of *otcR* mutant |
| OtcR-up-R2 | AAAAG*CTGCAG*GATCCAGCGGCCGAACC (*Pst*I) |  |
| OtcR-down-F2 | AAAA*CTGCAG*CGGATCCAGACGCTAATGGC (*Pst*I) |  |
| OtcR-down-R2 | CCG*GAATTC*AAGTAGTCGTAGAGGTACGG (*Eco*RI*）* |  |
| ∆OtcR-Test2-F | CAGAGGGACATATTGCCGCC |  |
| ∆OtcR-Test2-R | TCATGACCTGGTTCCTGCGC |  |
| PotcR-F | GGA*AGATCT*GACGCCTGACCGTACGAAGACG (*Bgl*II) | Construction of the complementary strain |
| PotcR-R | CTAG*TCTAGA*CTCCCAGATCGGCCAGTGAGG (*Xba*I) |  |
| Gfp-F | **TGAGCTACAATCAATACTCGATTAGGGATCC**GATTTCTGGAAACTAGAAGGAGG | green fluorescence gene (*gfp*) |
| Gfp-R | **AGCGGCCCTGGTTCCTTCTTGTTGTCT**AGAGAGTCACTAAGGGCTAACTAAC |  |
| PoxyA-gfp-F | **AGAAGTAGGGAAAACGGGGATGTTGAGATCT**GTATCTGGCCGAGGACATTCC | P*oxyA* promoter |
| PoxyA-gfp-R | **TTCCTCCTTCTAGTTTCCAGAAATCGGATCC**TGAGTCTCCTCTGCCGTCGTCG |  |
| PoxyI-gfp-F | **AGTAGGGAAAACGGGGATGTTGAGATCT**GGCCTTGTCCTCCGGTGTTGTG | P*oxyI* promoter |
| PoxyI-gfp-R | **TCCTCCTTCTAGTTTCCAGAAATCGGATCC**ACGACTCTCCTCTCCGGCG |  |
| PoxyI1*-F | **CGGACCGACCACGGCTCGAAGAACGGTCG**TAGACCGGCTGCCACGCTCACG | P*oxyI*1* promoter |
| PoxyI1*-R | **ACGACCGTTCTTCGAGCCGTGGTCGGTC** |  |
| PoxyI2*-F | **GATCACGGACCGACCACGGCTCGAAGAACGGAGGT**AGACCGGCTGCCACGCTCACG | P*oxyI*2* promoter |
| PoxyI2*-R | **ACCTCCGTTCTTCGAGCCGTGGTCGGTCCGTGATC** |  |
| PoxyJ-gfp-F | **GAAGTAGGGAAAACGGGGATGTTGAGATCT**ATACGACTCTCCTCTCCGGC | P*oxyJ* promoter |
| PoxyJ-gfp-R | **CTCCTTCTAGTTTCCAGAAATCGGATCC**GGCCTTGTCCTCCGGTGTTGTG |  |
| PoxyR-gfp-F | **GAAGTAGGGAAAACGGGGATGTTGAGATCT**ACACGCTCCTCGGATCATCAG | P*oxyR* promoter |
| PoxyR-gfp-R | **CCTCCTTCTAGTTTCCAGAAATCGGATCC**GAAGCGTCCTCGGGGTCCGTAG |  |
| PoxyS-gfp-F | **GAAGTAGGGAAAACGGGGATGTTGAGATCT**GAAGCGTCCTCGGGGTCCGTAG | Po*xyS* promoter |
| PoxyS-gfp-R | **TTCCTCCTTCTAGTTTCCAGAAATCGGATCC**ACACGCTCCTCGGATCATCAGTGG |  |
| SF14-F | **GATCCCCGGGGACCTGCAGGTCGACTCTAGCTA**GGCCTTGACCTTGATGAGGCG | Assembly of SF14-driven *otcR* |
| SF14-R | **ATGGGTCCTCCTGTGGAGTGGTTCTGTGGATCCCTAATCGAGTATTGATTGTAG** |  |
| Gib-otcR-F1 | **ACAGAACCACTCCACAGGAGGACCCATATG**GACTTCAAGGCACTCGG |  |
| Gib-otcR-R1 | **CTCACCGCGACGTATCGGGCCCTGGCCAGCTAG**GAATTCTCAAGACGCCGACC |  |
| OtcR-F | CGC*GGATCC*ACAGAACCACTCCACAGGAGGACCCATATGGACTTCAAGGCACTCGG (*Bam*HI) | Construction of pSF14-otcR |
| OtcR-R | CTAG*TCTAGA*TGTGTGGAATTGTGAGCGG (*Xba*I) |  |
| Gib-PotcR-F | **CCCGGGGACCTGCAGGTCGACTCTAGCTAG**GACGCCTGACCGTACGAAGACG | Assembly of pOtcR |
| Gib-PotcR-R | **CTCACCGCGACGTATCGGGCCCTGGCCAGCTAG**TCCCAGATCGGCCAGTGAGGC |  |
| Gib-otcR-F2 | **GTGAAATTGTTATCCGCTCACAATTCCACACAT**GCCTTGACCTTGATGAGGCGG | Assembly of pSF142-otcR and pSF143-otcR |
| Gib-otcR-R2 | **CTTGAAGCGGCCCTGGTTCCTTCTTGTTGTCTAGA** |  |
| TestF2 | GTGTTTGTGCGGCTTGAAGGGAAG | Identification of the transformants |
| TestR2 | ACCTCCAGGTTGGCCCAGGAGGACG |  |

***** The introduced restriction sites are shown in italics; the optimal ribosomal binding site (RBS) was introduced at the 5’ of *otcR* and is underlined; the overlaps sequences used for Gibson assembly are in bold.
